# Conjugation of an scFab domain to the oligomeric HIV envelope protein for use in immune targeting

**DOI:** 10.1371/journal.pone.0220986

**Published:** 2019-08-20

**Authors:** Hannah A. D. King, Christopher A. Gonelli, Kirsteen M. Tullett, Mireille H. Lahoud, Damian F. J. Purcell, Heidi E. Drummer, Pantelis Poumbourios, Rob J. Center

**Affiliations:** 1 Disease Elimination, Burnet Institute, Melbourne, Victoria, Australia; 2 Department of Microbiology and Immunology at the Peter Doherty Institute for Infection and Immunity, University of Melbourne, Melbourne, Victoria, Australia; 3 Infection and Immunity Program, Monash Biomedicine Discovery Institute and Department of Biochemistry and Molecular Biology, Monash University, Melbourne, Victoria, Australia; 4 Department of Microbiology, Monash University, Melbourne, Victoria, Australia; University of Massachusetts Medical School, UNITED STATES

## Abstract

A promising strategy for the enhancement of vaccine-mediated immune responses is by directly targeting protein antigens to immune cells. Targeting of antigens to the dendritic cell (DC) molecule Clec9A has been shown to enhance antibody affinity and titers for model antigens, and influenza and enterovirus antigens, and may be advantageous for immunogens that otherwise fail to elicit antibodies with sufficient titers and breadth for broad protection, such as the envelope protein (Env) of HIV. Previously employed targeting strategies often utilize receptor-specific antibodies, however it is impractical to conjugate a bivalent IgG antibody to oligomeric antigens, including HIV Env trimers. Here we designed single chain variable fragment (scFv) and single chain Fab (scFab) constructs of a Clec9A-targeting antibody, expressed as genetically fused conjugates with the soluble ectodomain of Env, gp140. This conjugation did not affect the presentation of Env neutralising antibody epitopes. The scFab moiety was shown to be more stable than scFv, and in the context of gp140 fusions, was able to mediate better binding to recombinant and cell surface-expressed Clec9A, although the level of binding to cell-surface Clec9A was lower than that of the anti-Clec9A IgG. However, binding to Clec9A on the surface of DCs was not detected. Mouse immunization experiments suggested that the Clec9A-binding activity of the scFab-gp140 conjugate was insufficient to enhance Env-specific antibody responses. This is an important first proof of principle study demonstrating the conjugation of a scFab to an oligomeric protein antigen, and that an scFab displays better antigen binding than the corresponding scFv. Future developments of this technique that increase the scFab affinity will provide a valuable means to target oligomeric proteins to cell surface antigens of interest, improving vaccine-generated immune responses.

## Introduction

Immune targeting is a promising strategy to enhance the immunogenicity of antigens which fail to elicit sufficient immune responses in their native state. One such antigen is the surface envelope protein (Env) of HIV, which is found on the viral membrane as a trimer of gp120-gp41 heterodimers. When expressing Env as a soluble protein for use in vaccinations, it is truncated immediately N-terminal to the transmembrane domain, creating a soluble construct known as gp140 [1], which is often stabilized by removal of the gp120-gp41 cleavage site (uncleaved gp140) [1, 2] or by the introduction of interdomain disulfide bonds (known as SOSIP) [3]. Immunization strategies for HIV have thus far failed to elicit broadly protective antibody responses, with the single effective HIV-1 vaccine trial in humans to date (RV144), reporting only 31% efficacy via Fc-mediated mechanisms [4]. A more highly efficacious vaccine for HIV will likely require antibody-mediated broad neutralization of this highly variable virus. Antibodies capable of mediating such neutralization are known as broadly neutralizing antibodies (bNAbs), and are directed against conserved sites on Env, enabling the neutralization of the majority of HIV strains. The elicitation of bNAb responses is observed in approximately 20% of those infected with HIV [5–7]. Passive immunization studies in non-human primates have shown bNAbs can protect against infection, at concentrations likely achievable by vaccination [8–13] (and references therein).

Many HIV bNAbs display characteristics that distinguish them from non-neutralizing antibodies (nNAbs). One such characteristic is the very long heavy-chain complementarity-determining region 3 (CDRH3) [14]. bNAbs often develop following years of infection [5–7], accumulating a large number of mutations relative to germline genes that confer their broad neutralizing capabilities [15], likely a result of prolonged antigen stimulation. These levels range from 20% changes in variable-domain amino acids for PG9 and PG16 [16], to 31% for 2G12 [17, 18] and 46% for VRC01 [19]. In contrast, amino acid mutations in the variable regions of other antibodies, such as those elicited by anthrax or influenza vaccines, or influenza infection, are typically between 5–15% relative to germline [20]. Previous studies analyzing less broad-acting and/or potent HIV-1 Env-specific antibodies reported a mutation rate of 5–10% [21, 22], indicating that a high level of affinity maturation is a specific characteristic of bNAbs. A very high level of affinity maturation is not an absolute requirement for broad neutralizing activity [23, 24], however many bNAbs contain mutations in uncommon sites within their variable regions [25], and the degree of neutralization breadth has been shown to correlate with affinity maturation [26].

The elicitation of bNAbs by vaccination will likely require enhanced levels of affinity maturation. This process occurs within germinal centers, and involves somatic hypermutation which alters the affinity of antibody for antigen coupled with clonal selection of those antibodies with a higher affinity for antigen, at the expense of clones encoding lower affinity antibodies [27, 28]. During HIV infection, an individual’s antibody response drives extensive mutation of Env, resulting in a co-evolution of the immune system attempting to control viral replication and escape of the virus from this immune response. This process, over a period of years, drives a high degree of antibody affinity mutation, and the elicitation of bNAbs in some HIV-positive patients. In order for vaccine-mediated responses to elicit high levels of affinity maturation, it is likely that the germinal center reaction will need to be accelerated. Immunization trials in non-human primates have shown the elicitation of neutralizing antibodies is associated with the enhanced formation of germinal centers and T follicular helper cell (Tfh) responses [29, 30].

A potential strategy to enhance the affinity maturation elicited during vaccination is the targeting of Env to the immune cells involved in this process. This strategy has been attempted for Env protein antigens previously, with the JR-FL strain of Env being targeted to the B cell receptors BCMA and TACI, via conjugation of Env to their shared ligands BAFF (B-Cell Activating Factor) and APRIL (A PRoliferation-Inducing Ligand), or to CD40 via CD40 ligand (CD40L) [31]. These targeting strategies enhanced the expression of activation-induced cytidine deaminase (AID), required for affinity maturation, with targeting via APRIL eliciting the highest anti-Env antibody titers as well as some neutralization of Tier 1 viruses [31]. Targeting of Env to Complement Receptor 2 (CR2) has also been extensively trialed, leading to higher antibody titers and a faster onset of affinity maturation [32–35], and enhanced neutralizing activity [36] compared to untargeted Env.

Another potential strategy for the elicitation of enhanced affinity maturation, which to our knowledge has not previously been assessed for Env, is the targeting of antigen to Clec9A. Clec9A (also known as DNGR-1) is a C-type lectin-like molecule [37–39], whose natural ligand is filamentous actin exposed on apoptotic cells [40, 41], with Clec9A binding initiating an adaptive immune response following apoptosis. Expression of Clec9A defines the CD8α+-like (BDCA3+, cDC1 lineage DC) subset of dendritic cells (DCs) in mice and humans [42], with this subset being specialized for the cross-presentation of antigen via MHC I molecules to CD8+ T cells. DC-targeting has been evaluated extensively for cancer and protein antigens, by directing antigen to molecules including but not limited to DEC-205, DC-SIGN, and DCIR [43]. Anti-Clec9A antibodies have been used to target model antigens including ovalbumin and nitrophenol to Clec9A in a number of vaccination studies, as well as HIV Gag p24 [44] and influenza matrix protein and enterovirus p71 antigen [45]. Targeting of antigen to Clec9A leads to enhanced antigen presentation on the surface of DCs, thus eliciting CD8+ and CD4+ T cell proliferation [44, 46, 47]. The Clec9A-targeted antigen is also able to persist in serum for up to 4 days post-immunization, likely contributing to the development of a follicular response as indicated by the elicitation of Tfh, formation of germinal centers, enhanced antibody titers, increased B cell affinity and formation of B cell memory [45, 47–50]. Clec9A-targeting can also be effective in the absence of adjuvant [39, 47, 48, 50]. This suggests that targeting of Clec9A is a potential strategy to enhance antibody affinity maturation, which is likely necessary for the induction of bNAbs against HIV Env.

Immune targeting with Clec9A, as with many other immune targeting strategies, has previously been achieved via genetic fusion of antigen to an anti-Clec9A antibody. This method, while appropriate for monomeric antigens, presents difficulties when conjugating multimeric antigens such as Env. Instead, we decided to use single chain antibody constructs, which can be expressed as genetic fusions with each gp140 subunit of a trimer. The conventional single chain targeting strategy involves the use of single-chain variable fragments (scFvs), in which the variable regions of the antibody heavy and light chains are fused with a short linker peptide. The scFv approach has been extensively used for phage display, due to their high display levels facilitating a high throughput approach [51]. ScFv fragments have also been explored as therapeutics, for targeted drug delivery of cancer immunotherapy [52] or targeting of immunogens to specific cell types [53–55], among other applications. Single-chain Fab (scFab) are a less well-investigated single-chain antibody fragment design. ScFab have been used in phage display platforms and offer the advantage of higher stability in comparison to an scFv [56–59]. ScFab-like antibodies have mainly been used in the production of bi-, tri- and tetra-specific antibodies [60–66], have been less frequently expressed as a single antigen combining site [67] and have not been previously used for immune targeting strategies. Here, we developed a novel approach for attaching an scFab to an oligomeric protein whose geometry is not compatible with a bivalent IgG. The scFab domain/Env fusion protein was efficiently expressed and mediated enhanced binding of the target Clec9A antigen when compared to the more conventional scFv targeting moiety, although the affinity remained lower than that of the unconjugated intact IgG antibody.

## Methods

### Plasmids

**Fig 1:**
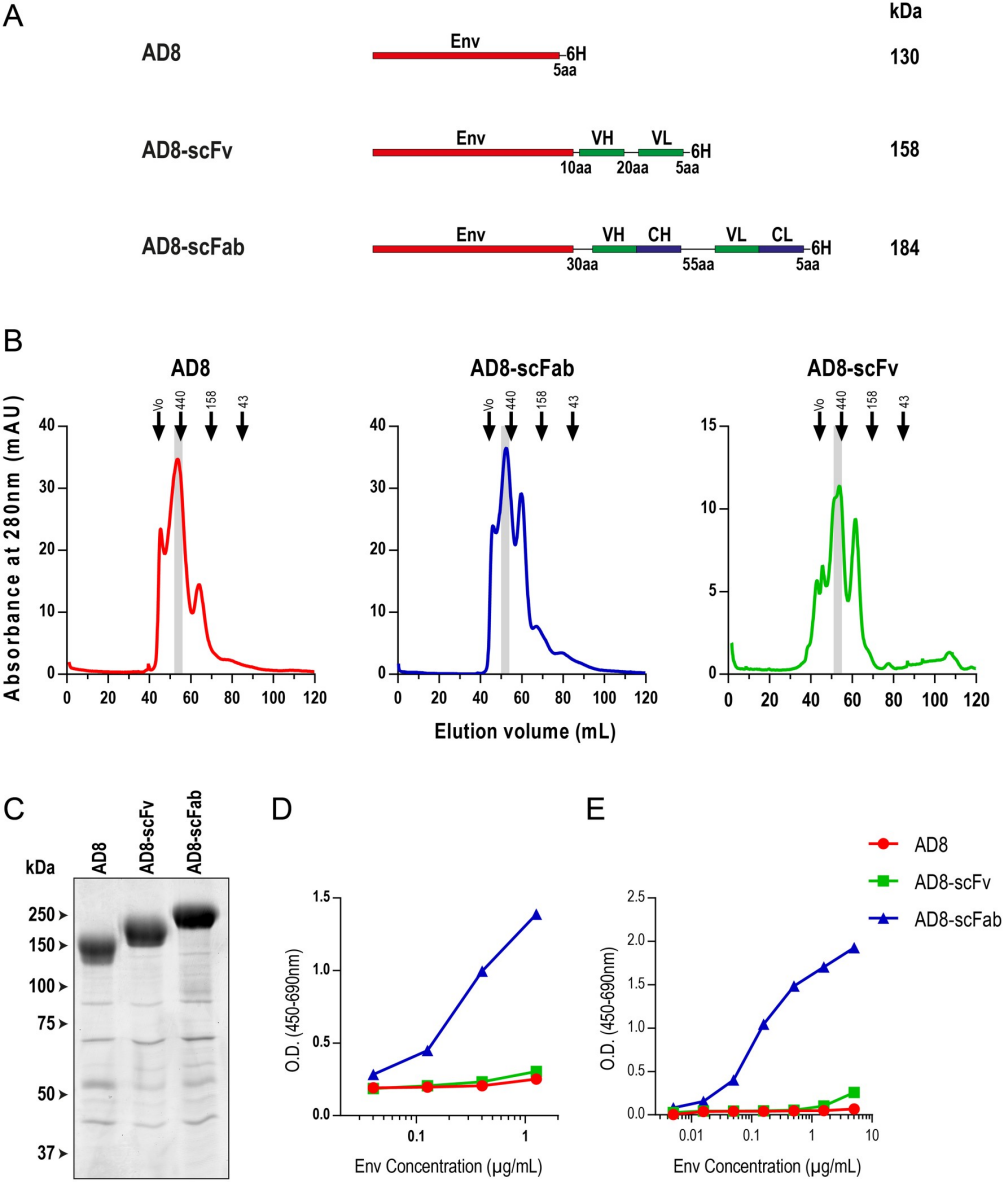
Design and Expression of Clec9A-targeting constructs. (A) Schematic representation of Env constructs with anti-Clec9A scFv and scFab domains. Env is an uncleaved AD8 gp140. The lengths of linkers between domains are given (amino acids = aa). These linkers are composed of serine/glycine repeats n(SGGG). VH = variable heavy, VL = variable light, CH = constant heavy and CL = constant light. A 6X HIS tag is represented by 6H. Approximate molecular weights are given besides each construct, based upon amino acid sequence and predicted occupancy of glycan sites, determined using the NetNGlyc 1.0 Server (Technical University of Denmark). (B) Preparative SEC traces of AD8, AD8-scFv and AD8-scFab. Samples were run on a 16/600 Superdex 200 SEC column and elution was measured using absorbance at 280nm. The void volume (V_0_) and elution of molecular weight standards are indicated by arrows, and putative trimer-containing peaks, pooled for further use, are shaded grey. (C) Coomassie blue stained purified trimeric proteins following SDS-PAGE. Samples (5μg) were run on an 8% SDS-polyacrylamide gel under reducing conditions then stained for total protein with Coomassie Blue R-250. Size standards used were Precision Plus Protein All Blue Standards (Bio-Rad). (D and E) An ELISA was performed to assess binding of Env in tissue culture supernatants prior to purification (D) or purified Env (E) to Clec9A. An ELISA plate was coated with recombinant Clec9A. Env was then added to the first well and diluted in a half log series. The loading of unpurified Env was determined by densitometry analysis of an anti-gp120 western blot, in comparison to purified Env of known concentration. Env binding was detected using pooled HIV+ sera. Due to limitations in the availability of recombinant Clec9A, these results were generated from a single experiment.

**Fig 2:**
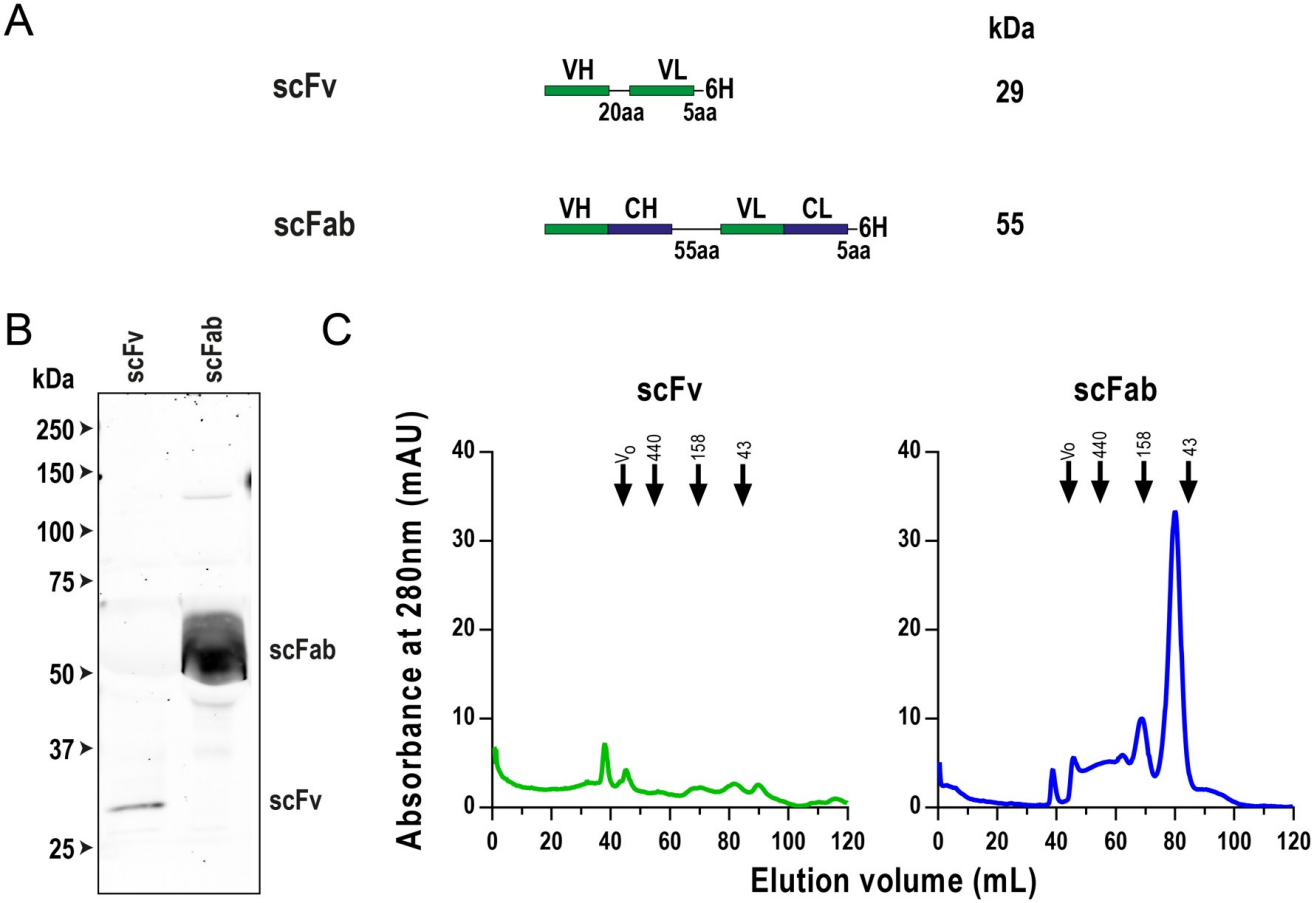
Expression of scFv and scFab constructs in isolation. (A) Schematic representation of anti-Clec9A scFv and scFab constructs. The lengths of linkers between domains are given (amino acids = aa). These linkers are composed of serine/glycine repeats n(SGGG). VH = variable heavy, VL = variable light, CH = constant heavy and CL = constant light. A 6X HIS tag is represented by 6H. Approximate molecular weights are given besides each construct, based upon amino acid sequence. (B) Western blot of purified proteins. Volume equalized samples were run on an 8% SDS-polyacrylamide gel under reducing conditions before being transferred to a nitrocellulose membrane. The gel was probed with a rabbit anti-6X HIS antibody and was imaged at 800nm. Size standards used were Precision Plus Protein All Blue Standards. (C) Preparative SEC traces. Protein was run on a 16/600 Superdex 200 pg SEC column and elution was measured using absorbance at 280nm. The void volume (V_0_) and elution of molecular weight standards are indicated by arrows.

The Clade B, R5-tropic AD8 Env [68] was expressed from the pN1-AD8-140 plasmid as described [69]. The pN1-AD8-140-scFv and pN1-AD8-140-scFab plasmids were created by inserting either an scFv or scFab fragment of the 10B4 IgG2a rat anti-mouse Clec9A antibody [39] into the region corresponding to the C-terminal end of gp140. The resulting protein encoding domains of these plasmids are shown in Fig 1A. The plasmids expressing the scFv and scFab alone, p-scFv and p-scFab were created by inserting the scFv and scFab DNA sequences with a tissue plasminogen activator leader sequence into pcDNA3.1 (Invitrogen). The resulting protein encoding domains of these plasmids are shown in Fig 2A. To express cell-surface Clec9A, a pClec9A-EGFP plasmid was used, which was created by replacing the G418 resistance gene into a plasmid expressing full length Clec9A [39] with an EGFP gene, such that Clec9A was expressed from a CMV promoter and EGFP from an Internal Ribosome Entry Site (IRES) to enable gating on transfected Clec9A-expressing cells. The AD8 SOSIP.v4 and SC45 SOSIP.v4 proteins were expressed from a CMV-promoter driven pcDNA3 plasmid described previously [70]. The Env ectodomain segment was linked to a C-terminal 6X HIS tag with a GGGGS linker, with the addition of the SOS and I559P mutations [3], an RRRRKR cleavage site and the stabilising L543N, E64K and A316W mutations [71]. The BG505 protein was similarly modified but did not contain the L543N, E64K and A316W mutations. The MW965 uncleaved gp140 was expressed from the same pN1 plasmid backbone as the AD8 uncleaved gp140 protein [72].

### Cell culture

Freestyle 293-F cells (293-F) cells were maintained in Freestyle 293 Expression Medium (Invitrogen). Cells were cultured at 37°C in 8% (v/v) CO_2_ in Corning Erlenmeyer flasks with agitation (relative centrifugal force approximately 5 x g). Embryonic Kidney (HEK293T) cells (American tissue culture collection CRL-11268) were maintained in Dulbecco’s Modified Eagles Medium supplemented with 10% (v/v) Fetal Bovine Serum, 2mM HEPES, 2mM L-glutamine, 100μg/mL Gentamycin and 1μg/mL Minocycline.

### Expression and purification of protein constructs

To produce protein, plasmids were transfected into 293-F cells using 293fectin (Invitrogen) according to the manufacturer’s recommendations or into 293T cells using polyethylenimine [73]. 24 hours post-transfection, 0.5% (w/v) lupin peptone and 0.02% (w/v) Pluronic F-68 were added to the cells. Protein-containing supernatants were harvested following 3–5 days of incubation by centrifugation at 1,500 x g for 5 minutes to remove cells, followed by filtration through a 0.45μm filter.

Proteins were purified from supernatant using Talon metal affinity resin (Clontech Laboratories) with the exception of MW965 which was purified using lentil lectin affinity chromatography (GE Healthcare). Eluates were concentrated and subjected to size exclusion chromatography (SEC) using a 16/60 Superdex 200 pg column and an AKTA Pure liquid chromatography system (GE Healthcare) at a flow rate of 1ml/min with PBS as the buffer. The column was calibrated by measuring the elution of protein standards of known molecular weight: blue dextran (void), ferritin (440 kDa), aldolase (158 kDa) and ovalbumin (43 kDa). Fractions containing Env trimers, or antibody fragments were pooled, concentrated and filter sterilized using a 0.22μm filter.

Proteins were separated on the basis of size in SDS polyacrylamide gels. Specific proteins were visualized via Western blotting. Electrophoresed proteins were transferred onto a nitrocellulose membrane. The membrane was then blocked using block buffer (5% (w/v) skim milk powder, 0.05% (v/v) Tween in PBS) prior to antibody staining with an anti-6X HIS epitope tag antibody (Rockland Immunochemicals), and then rabbit IgG (H&L) IRDye800CW antibody (Rockland Immunochemicals) at 0.5μg/mL. The gels were imaged using a Li-COR Odyssey imager at 800nm. Alternatively, to assess total protein present in a sample, SDS gels were stained using 0.25% (v/v) Coomassie brilliant blue R-250.

### Binding of Env constructs to bNAbs by ELISA

To measure the binding of bNAbs to the Env constructs, 96-well plates were coated with 100ng/well AD8 gp140 (or a molar equivalent of AD8-scFab) overnight at 4°C in 100mM NaCl and 20mM Tris, pH 8.8. All subsequent steps were performed at room temperature. Wells were washed sequentially in PBS/0.05% Tween 20 and PBS and then blocked with block buffer (PBS/0.05% Tween 20/5% skim milk powder) for 1 hour, with all following steps performed in block buffer. The primary antibodies used were the bNAbs PGT121, PG9 and VRC01, which were expressed in 293-F cells by transfection with an equimolar ratio of antibody heavy and light chain genes, with antibody purified from the supernatant using Protein G sepharose. The bNAb 2G12 was kindly provided by Bruce Wines (Burnet Institute) and was originally provided by John Moore (Weill Cornell medical college), and the 2F5 antibody was obtained through the NIH AIDS Reagent Program, Division of AIDS, NIAID, NIH from Polymun Scientific [74]. The primary antibodies were titrated on the ELISA plate, and incubated for one hour. Following washing, the secondary anti-human antibody (Polyclonal Rabbit Anti-Human IgA, IgG, IgM, Kappa, Lambda/HRP, Agilent Technologies) was added. After a one-hour incubation, wells were washed and developed by the addition of 3,3’,5,5’-Tetramethybenzidene dihydrochloride and absorbance measured at 450nm, with the background at 690nm subtracted.

### Binding of Env constructs to Clec9A

#### ELISA to measure Clec9A-binding

To measure the binding of Env constructs to Clec9A, a capture ELISA was used. Soluble Clec9A ectodomain [41] was coated onto 96-well plates at 100 ng/well overnight at 4°C in 100mM NaCl and 20mM Tris, pH 8.8. Plates were washed and blocked, and subsequent steps were performed at room temperature and samples diluted with block buffer, as described for the bNAb-binding ELISA. The Env protein was then titrated and incubated for two hours to allow capture of recombinant anti-Clec9A-Env constructs. The primary antibody, a 1:1000 dilution of a pooled human anti-HIV serum (kindly provided by Prof. Anthony Kelleher, Kirby Institute, UNSW, Australia), was then added and was incubated for 1 hour. The secondary antibody and development steps are as described for the bNAb-binding ELISA above.

#### Flow cytometric analysis of Clec9A binding

293T cells were transfected with pClec9A-EGFP using Fugene HD. 48 hours later cells were harvested by disrupting the monolayer via repeated pipetting. The cells (1x10^6^ cells in 100μL/sample) were stained with 10μg/mL uncleaved AD8 gp140 (or the molar equivalent of AD8-scFv or AD8-scFab) or 10μg/mL 10B4 anti-Clec9A antibody, followed 5μg/mL of the secondary antibodies, either PGT121-CF647 (PGT121 antibody labelled with CF647 using a Mix-n-Stain CF647 Antibody Labelling Kit (Sigma Aldrich)) for the Env-containing samples, or anti-rat CF647, for the 10B4 sample. Prior to analysis cells were incubated with the live/dead stain Fixable Viability Dye eFluor 506 (Thermo Fisher Scientific), then fixed using 1% paraformaldehyde in PBS. Analysis was performed on a BD LSR II flow cytometer (Becton, Dickinson and Company) with fluorescence compensated as appropriate. Plots were created using the FlowJo v9 software. Control samples included cells that had been incubated with secondary antibody only, as well as unstained cells.

The DC cell line Mutu DC 1940 [75] was stained with the Env constructs as described for the 293T cells. Binding of a biotinylated anti-Clec9A monoclonal antibody (clone 10B4) and an isotype control (rat IgG clone eBR2a (eBioscience)) were detected using Streptavidin PE. Propidium iodide was used to label dead cells.

### Immunization of experimental animals

Female Balb/C mice were used for all experiments. At the beginning of experiments mice were 6–8 weeks old. Mice were housed under specific pathogen-free conditions at the Peter Doherty Institute for Infection and Immunity Bioresources Facility. Mice were kept in groups of 5 mice per box, with free access to food and water in a clean room with a 12-hour day/ night cycle, and a temperature between 20–22°C. Mice were monitored for adverse events including those requiring euthanasia one hour and 24 hours following all procedures, with regular biweekly monitoring. Ethics for experiments using mice were obtained from the University of Melbourne Animal Ethics Committee (Ethics I.D. 1312863).

Mice were vaccinated twice with 2μg Env (or molar equivalent targeted antigen) at 0 and 4 weeks, then again with 5μg at 23 weeks. A total volume of 200μL was injected both intraperitoneally and subcutaneously, with the vaccine dose split equally between these two sites. The adjuvant Addavax (InvivoGen) was used at a 1:1 volume ratio with antigen. Sera were collected by tail vein bleeding. For terminal sera collection, mice were culled by CO_2_ asphyxiation, then blood collected by heart puncture. Untreated blood was incubated at room temperature for 1 hour to allow clotting, then centrifuged at 8000 x g for 15 minutes at 18°C. The sera were then collected and stored at -20°C.

### Measurement of immune responses

The presence and titer of Env-specific antibodies in murine sera was determined by D7324-capture ELISA as previously described [69]. D7324 is a sheep antibody with reactivity to a linear epitope in the C-terminal region of gp120 used to capture the AD8 gp140 protein. When binding to Envs of different clades was measured, Env was directly coated onto the ELISA plates at 100ng/well. To measure anti-scFab immune responses, the same ELISA format was used, however the antigen (10B4 antibody) was directly coated onto the ELISA plate at 50ng/well. The secondary antibody used to assess mouse immunoglobulin binding was a polyclonal rabbit anti-Mouse immunoglobulins/Horse Radish Peroxidase (HRP) antibody (Agilent Technologies).

### Statistical analysis

Statistics were assessed using GraphPad Prism v7 software. Where relevant, sample mean is indicated, with error bars representing standard error of the mean (SEM). Statistical analyses of results were performed using a Kruskal-Wallis test with Dunn’s multiple comparisons tests. When standard curves were plotted, a log(inhibitor) vs. response—Variable slope (four parameters) function was used. Correlations between different parameters were assessed using the non-parametric Spearman rank order correlation. Differences were deemed to be significant when p<0.05.

## Results

### Design of Clec9A-targeting constructs

In order to target Env to Clec9A+ DC, constructs directly fusing anti-Clec9A antibody fragments to Env were designed. These constructs used the AD8 strain of Env, containing mutations in the protease cleavage site to prevent cleavage and preserve the peptide bond linking gp120 and gp41. Translation of Env was terminated prior to the transmembrane domain, leading to expression of a soluble uncleaved Env, gp140.

While previous Clec9A targeting strategies have used anti-Clec9A antibodies (whole IgG) for targeting with monomeric antigen, the fusion of trimeric Env to a dimeric antibody would likely cause misfolding or misassembly of one or both moieties. Therefore, a single-chain variable fragment (scFv) and a single-chain Fab (scFab) of the rat anti-murine-Clec9A antibody 10B4, were introduced into the Env-expression plasmid (Fig 1A). The scFv, typically used for single chain targeting of antigens, was made by linking the variable regions of the antibody to Env. A 10 amino acid serine/glycine linker connected Env to the VH region, and a 20 amino acid linker connected VH to the VL region. An scFab was also assessed for potentially enhanced stability and affinity for the targeting moiety, and its design was based on a previously described method [59]. This used a linker length of 55 amino acids between the heavy and light chains, and a 30 amino acid linker between Env and the VH region. All linkers used were composed of serine and glycine residues n(SGGGG), chosen as they are small and highly flexible. The 30 amino acid linker between the scFab and Env was predicted to be 36Å in a relaxed format and 72Å in its fully extended state, with a linear distance between the Fab fragments of approximately 65Å [59]. This distance was chosen to allow the variable regions, which in the construct are located at the N-terminus of the Fab moiety, proximal to Env, to flex and be exposed on the exterior surface of the molecule. The native disulfide bond connecting the Fab light and heavy chains was retained to enhance stability [76].

### Expression of Clec9A-targeting Env proteins

The AD8, AD8-scFv and AD8-scFab were expressed in 293-F cells and were initially purified using Talon metal affinity resin, then further purified to isolate trimers by SEC on a 16/600 Superdex 200 column (Fig 1B). The traces obtained for these Env proteins indicated the presence of a number of different oligomeric species, as has been previously reported for this uncleaved AD8 construct [77], with the addition of the Clec9A-targeting domains not greatly affecting the oligomeric profile of the protein. Trimeric gp140 was selected from the fractions indicated (based on expected migration compared to the protein standards of known molecular weight) for use in further analysis.

The purified trimers were then analyzed by SDS-PAGE followed by staining with Coomassie Blue for total protein (Fig 1C). The main AD8 band was 150 kDa, whereas AD8-scFv and AD8-scFab bands appeared at ~200 kDa and ~250 kDa, respectively. While this is higher than the predicted molecular weight of these proteins (130, 158 and 184 kDa), this migration pattern was consistently observed. In each protein sample a number of less intensely staining, more rapidly migrating bands were also visible. These are likely contaminants which bind non-specifically either to the Talon metal affinity resin or to Env. As the amount of these proteins is low relative to Env, and is consistent between samples, we considered it unlikely that their presence affected downstream analyses.

### The AD8-scFab construct is able to bind recombinant forms of Clec9A

We compared the ability of unpurified (Fig 1D) or purified (Fig 1E) AD8-scFab and AD8-scFv to bind plate-bound Clec9A by capture ELISA. This showed that the AD8-scFab binds recombinant Clec9A with approximately 100-fold higher magnitude than does AD8-scFv, which displayed negligible binding above background. As expected, unmodified AD8 did not show any binding to Clec9A, validating that binding was mediated specifically by the targeting domains.

### The scFab, but not scFv, can be expressed in the absence of Env

The very low Clec9a binding activity of AD8-scFv relative to AD8-scFab led us to further characterize the scFv and scFab moieties in isolation. Constructs were therefore created to express scFv and scFab alone. These constructs contained identical antibody domains, internal linkers and 6HIS tags at their C terminus as the corresponding previously used constructs that were fused to the C-terminus of Env (Fig 2A).

These constructs were expressed on a small-scale in 293T cells and the supernatants were harvested from the cells following 48 hours incubation, then run on an SDS-PAGE gel with 6HIS-tagged proteins visualized by Western blotting (Fig 2B). A very faint band corresponding to the expected scFv molecular weight of ~30 kDa was observed, while a prominent band of ~60 kDa was visible in the scFab sample, consistent with the predicted 55 kDa molecular mass of this antibody fragment. These data suggested that the scFab was expressed with much greater efficacy than the scFv.

The scFab and scFv were then expressed on a larger-scale by transfection in 293-F cells. Following 4 days incubation the supernatant was harvested and purified using Talon metal affinity resin, followed by further purification by SEC using a 16/600 Superdex 200 pg column (Fig 2C). For the scFab, a peak observed at ~80 mL corresponds with the retention volume expected for a single scFab (~55 kDa). Several minor peaks were also present, indicating improperly folded and aggregated forms. For the scFv however, no single dominant peak corresponding in molecular weight to the scFv was observed. The series of small peaks could be improperly folded scFv or contaminating proteins. Overall, the data indicate that the scFv was expressed at very low levels in 293 cells in comparison to the scFab, possibly indicating that the former is unstable. This precluded direct comparisons of the scFv and scFab binding capacities for Clec9A by flow cytometry. This likely instability of the scFv may explain its inability to bind recombinant Clec9A when conjugated to Env. Consequently, further analysis of AD8-scFv was not performed.

### AD8-scFab retains full reactivity to bNAbs

**Fig 3:**
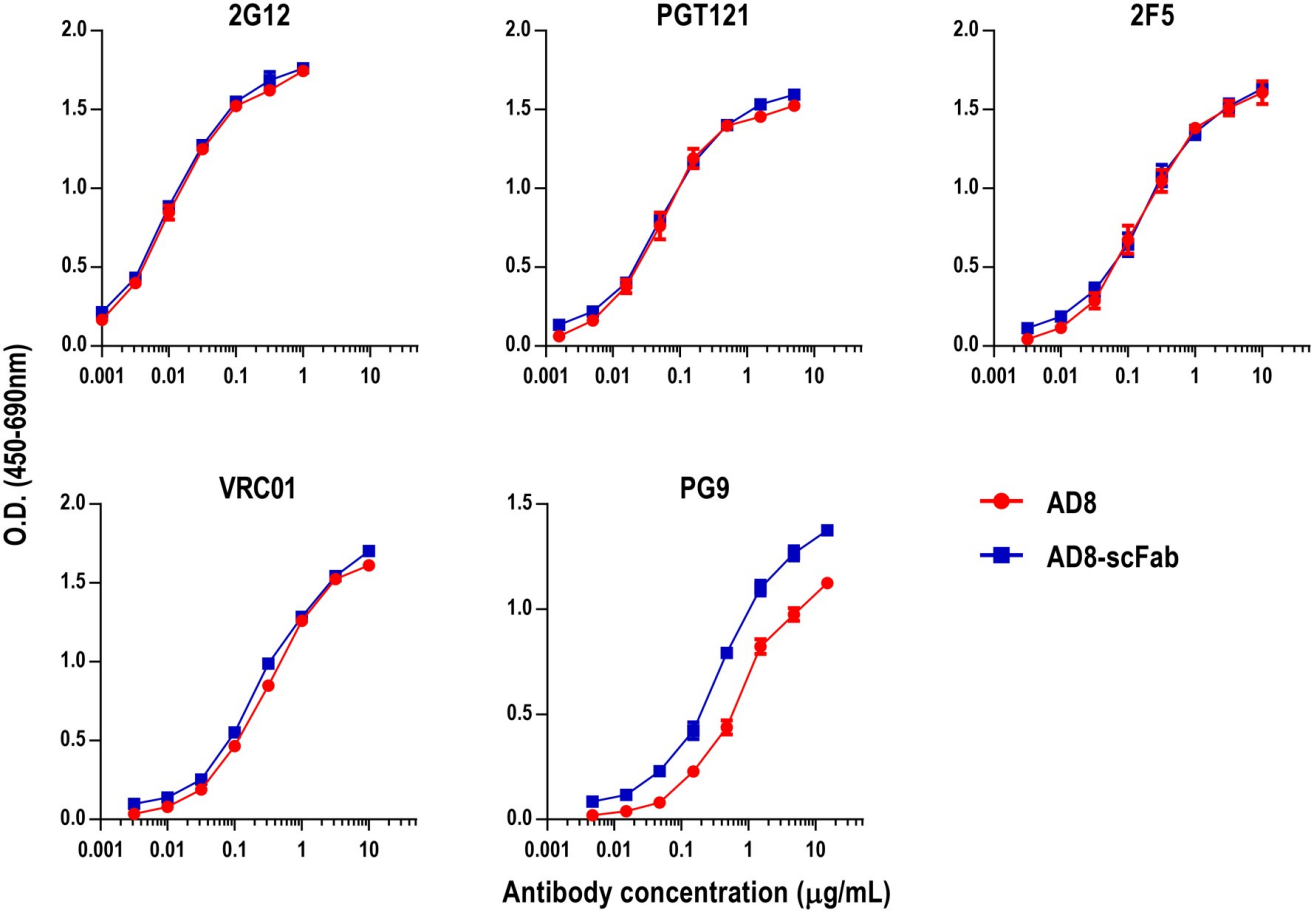
Binding of Clec9A-targeting constructs to bNAbs. Direct ELISAs were performed to assess the binding of Env constructs to bNAbs. ELISA plates were coated with 100ng/well AD8 gp140 (coating levels of AD8-scFab normalized based on molarity), then bNAbs were diluted in a half log series at the concentrations indicated. Graphs show the mean ± SEM of three independent experiments.

To assess whether the Env conformation was altered by the addition of the scFab domain, the binding of AD8-scFab to bNAbs was assessed by ELISA (Fig 3). The bNAbs were chosen as they bind epitopes in distinct regions of Env and are key epitopes against which we wish to elicit vaccine-mediated immune responses. 2G12 was included as its epitope is composed of only carbohydrates rather than protein and is less dependent on Env conformation [78], therefore it can be used to control for Env loading. The binding curves for AD8 and AD8-scFab overlap for 2G12, confirming the proteins were loaded equally. The antibodies PGT121, 2F5 and VRC01 bound AD8 and AD8-scFab equally. Surprisingly, the bNAb PG9, directed towards a quaternary epitope at the Env apex [79], bound AD8-scFab more strongly than AD8 by approximately 3-fold. This suggested that the scFab domain had some impact on Env conformation, however overall the effect was modest and the exposure of bNAb epitopes was maintained in AD8-scFab.

### Binding of Env-scFab to cell-surface forms of Clec9A

**Fig 4:**
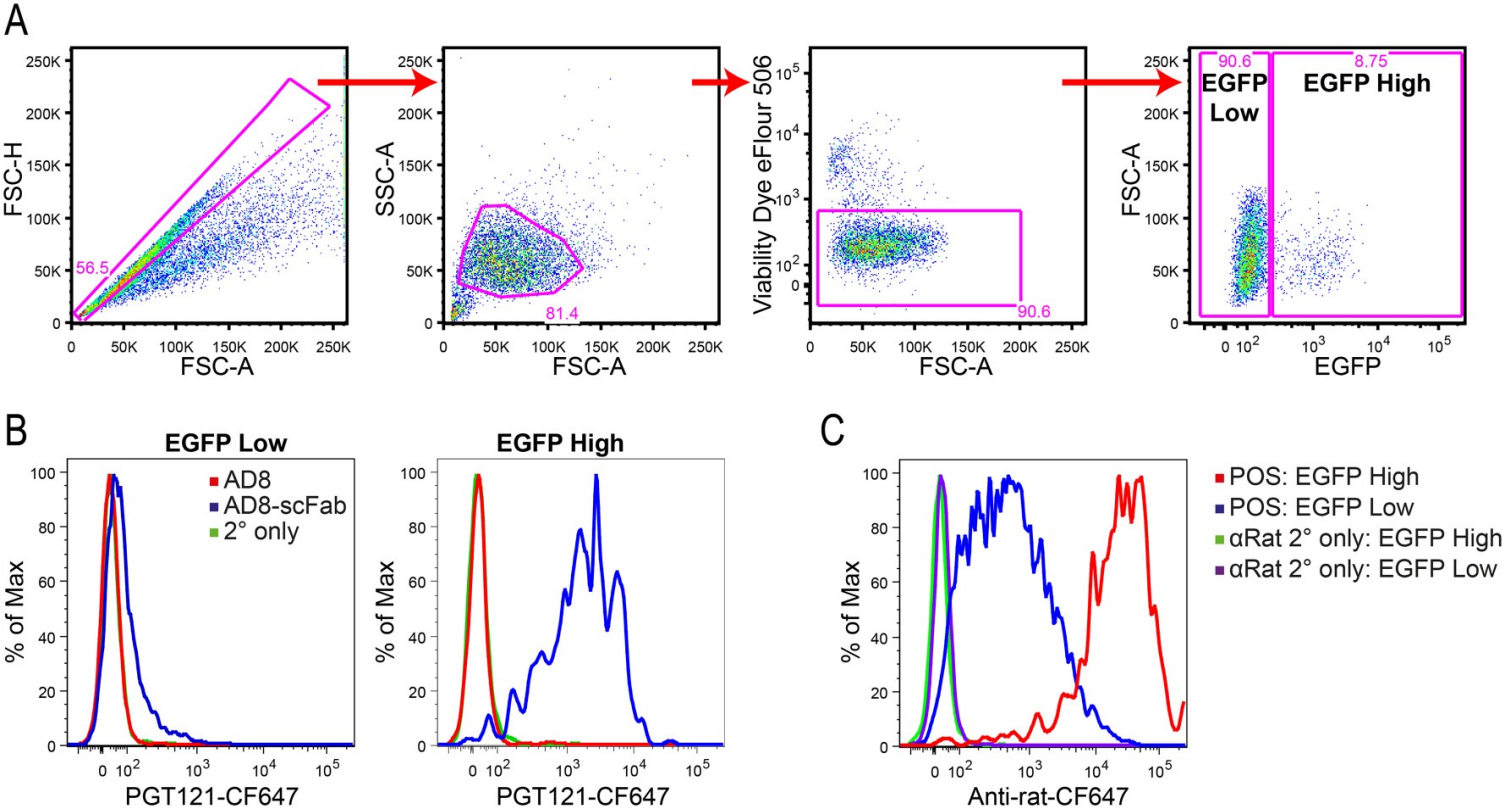
Binding of Clec9A-targeting constructs to cell surface Clec9A. (A) 293T cells were transfected with pClec9A-EGFP. Cells were incubated with Env constructs, and then fluorescently stained with PGT121-CF647. For a positive control of Clec9A expression, cells were stained with 10B4, followed by anti-rat-IgG2a-CF647. Cells were initially gated for single cells followed by selection of eFluor-506 negative cells to remove dead cells. Cells were then gated as being either EGFP High or EGFP Low. (B and C) Histogram showing CF647 fluorescence, indicative of Clec9A binding, on EGFP High and Low cells incubated with Env constructs (B) or Positive control 10B4 antibody (C). Cells were gated as described in A and curves were normalized to mode. Data shown are representative of 3 replicates.

To assess whether the AD8-scFab construct was able to target Clec9A when expressed on the cell surface, flow cytometry was performed. 293T cells were transfected with a plasmid expressing both membrane-bound Clec9A and EGFP through an IRES (pClec9A-EGFP). Initially, single cells were selected (Fig 4A), followed by exclusion of cellular debris based on forward and side scatter and dead cells by their binding to viability dye. The co-expression of EGFP with Clec9A enabled gating on transfected cells expressing high or low levels of EGFP as a surrogate marker for high or low Clec9A expression, respectively, in subsequent analysis. The binding of Env constructs to surface Clec9A was measured using the Env-specific PGT121-CF647 antibody (Fig 4B). The binding of AD8-scFab to Clec9A on the EGFP High cells is approximately 2 logs higher than that of AD8, which bound at a level equivalent to background (Fig 4B). The binding of the positive control antibody 10B4 to the EGFP High cells, detected using an anti-rat-CF647 antibody, was high, with an MFI between 10^4^−10^5^ (Fig 4C). The 10B4 positive control antibody was able to bind the EGFP Low cells, while the AD8-scFab binding was low and similar to that of AD8 alone (Fig 4B and 4C). The binding of the anti-Env secondary antibody only control was equivalent to AD8 binding, indicating that 10B4 and AD8-scFab mediate specific binding to Clec9A-expressing cells (Fig 4B). The affinity of the AD8-scFab for Clec9A was less than the full antibody, indicated by its inability to bind to the EGFP Low cells, which expressed low levels of Clec9A. It should also be noted that although the binding of 10B4 gives a higher signal than that of AD8-scFab, the secondary antibodies will have different binding affinities for their respective targets and/or differing CF647 intensities, meaning a direct comparison of the Clec9A binding between samples stained with different secondary antibodies may not be representative of true binding efficiency.

The binding of the Env-scFab to a DC cell line constitutively expressing Clec9A, Mutu DC 1940, was then investigated by flow cytometry. While the full anti-Clec9A 10B4 antibody displayed binding to this cell line, the Env-scFab did not detectably bind, with an MFI equal to background levels (S1 Fig). This DC cell line expresses lower levels of Clec9A, and so is more biologically relevant to levels of Clec9A in vivo when compared to Clec9A expression on transfected cells. The data suggest that Env-scFab requires a high density of surface Clec9a molecules for efficient binding.

### Immunogenicity of AD8-scFab

**Fig 5:**
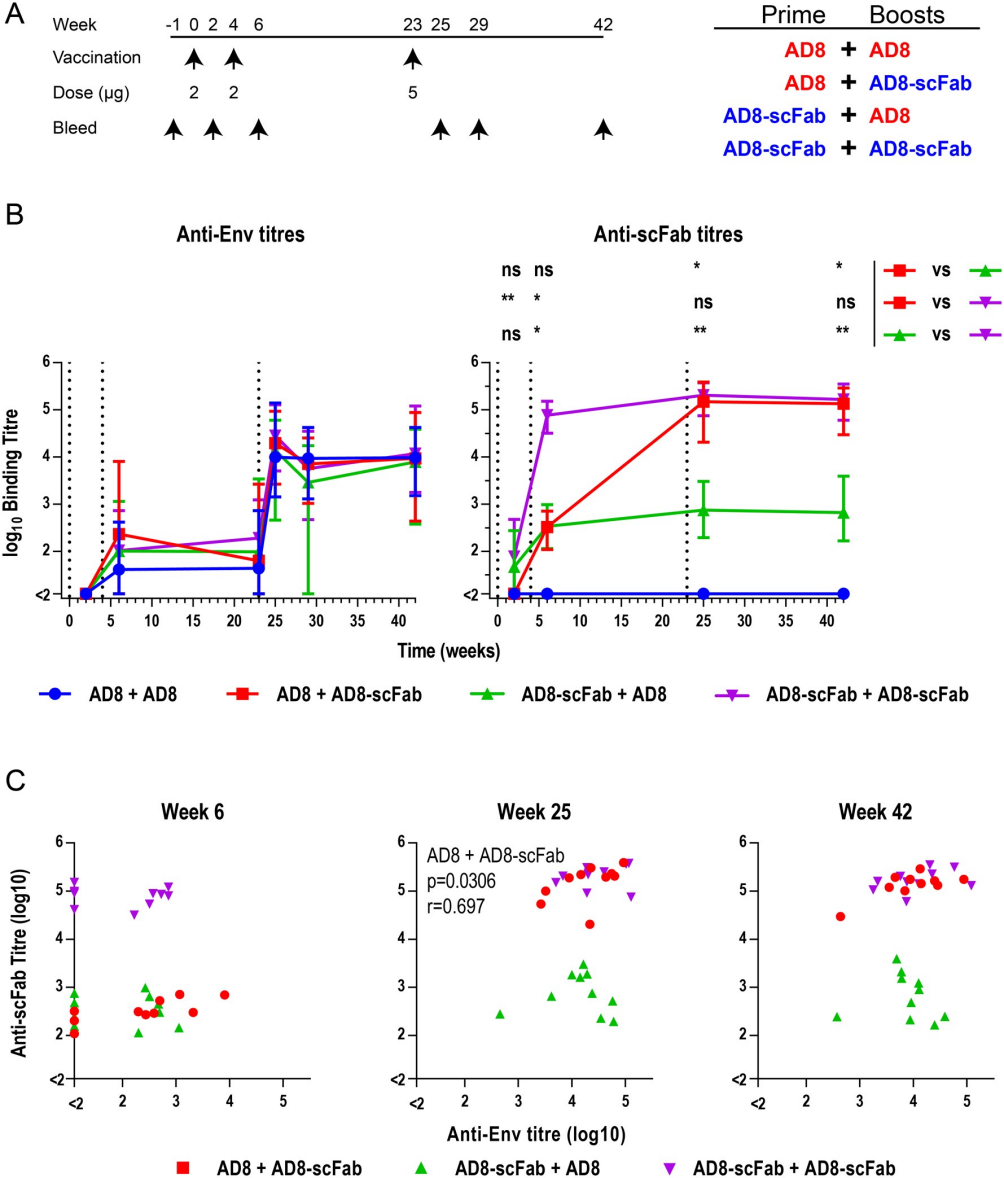
Antibody titers elicited by vaccination with Env-scFab. (A) Schematic of vaccination schedule and groups. (B) Anti-Env and anti-scFab titers in sera measured by ELISA. For anti-Env titers, 5μL/well of concentrated AD8 gp140 293T supernatant was captured onto the ELISA plate using D7324, then mouse sera were half-log serially diluted from an initial 1:100 dilution. For anti-scFab titers ELISA plates were coated with 10B4, then mouse sera were diluted in a half-log series. Titers were defined as the dilution at which the ELISA curve was equal to 3x the average of the adjuvant only group. Graph shows the mean of individual titers within each vaccination group, error bars represent the range and the vertical dotted lines indicating vaccination times. Significance was assessed using a Kruskal Wallis test, followed by a Dunn’s post-test, with * p<0.05, ** p<0.01 and ns not significant. (C) Correlations of anti-scFab and anti-Env titers at indicated time points. Correlations were assessed for groups that received the AD8-scFab immunogen using a Spearman correlation.

To assess whether the low levels of Clec9A binding mediated by the AD8-scFab were sufficient to mediate immune enhancement, we assessed the immunogenicity of AD8-scFab compared to AD8 gp140 by vaccination of mice. Mice were vaccinated three times: at weeks 0 and 4 with 2μg AD8 gp140 and at week 23 with 5μg AD8 gp140, or an equimolar amount of AD8-scFab. A vaccination strategy was employed to assess the effects of priming or boosting with AD8-scFab, with one group receiving an AD8 gp140 prime followed by the two boosting immunizations with AD8-scFab and another group receiving an AD8-scFab prime followed by two AD8 gp140 boosts. Additionally, two groups of animals were primed and boosted with homologous AD8-scFab or AD8 gp140 antigen (Fig 5A). A fifth group was vaccinated with the adjuvant, Addavax, in the absence of Env for use as a negative control. Sera were collected from mice on the weeks indicated in Fig 5A. The anti-Env titers were assessed by D7324 capture ELISA (Fig 5B, left). Anti-Env responses were observed following the second vaccination and at subsequent time-points. Prior to the third vaccination the anti-Env titers were low and inconsistent, with some animals not having titers above background. Responses increased 2 weeks after the final dose at week 25, with positive titers in all animals. The responses decreased slightly at 29 weeks, then remained relatively consistent until 42 weeks. No statistically significant differences between experimental groups were observed in anti-Env titers at any time point. At week 23, the highest responses appeared to be elicited in the group that received two doses of AD8-scFab, although this trend decreased when all responses were boosted to a higher magnitude. Additionally, the breadth of antibody responses was measured to determine if this was enhanced by Clec9A-targeting. An ELISA assessing the sera responses to Envs SC45 SOSIP.v4.1 (clade B Transmitted/Founder), BG505 SOSIP (clade A) and MW965 uncleaved gp140 (clade C) revealed negligible differences in binding to these different Envs between the vaccine groups (S2 Fig), indicating AD8-scFab did not affect the cross reactivity of the antibodies elicited. Overall the data suggest that the AD8-scFab binding to Clec9A was likely not sufficient to induce enhanced antibody titers in this immunization trial.

We hypothesized that the scFab domain may itself be immunogenic in vaccinated mice as it is derived from a rat antibody. The reactivity of sera to the full 10B4 IgG was therefore assessed in a direct ELISA (Fig 5B, right). After the first vaccination, the groups that received AD8 displayed no reactivity, while the groups that received AD8-scFab displayed modest and inconsistent titers. Following the first boost however, the group that received two doses of AD8-scFab elicited anti-10B4 titers of ~10^5^, while the animals which had received one dose of AD8-scFab (either primed or boosted) displayed titers of ~10^2.5^. The group that only received AD8 had no anti-rat antibody responses at any time point, confirming the specificity of the assay. Following the third vaccination, animals that had received two doses of AD8-scFab elicited similar binding titers as those receiving only the AD8-scFab immunogen, suggesting that the anti-scFab titers plateau at about 10^5^ in this system. The group that was only primed with AD8-scFab retained titers of ~10^2.7^ from week 5 onwards.

To assess whether the strong immunogenicity of the scFab domain was impacting upon the anti-Env antibody titers elicited, we investigated whether there was any correlation between the anti-Env and anti-scFab titers within groups. To achieve this, a Spearman correlation was performed on the anti-Env and anti-rat IgG titers obtained at weeks 6, 25 and 42 (Fig 5C). In general, no positive or negative correlations were observed. A small positive correlation between anti-Env and anti-scFab titers was observed for week 25 sera from the group which was primed with AD8 and boosted with AD8-scFab, but not for other groups or other time points.

## Discussion

Here we demonstrate for the first time the conjugation of an scFab domain to an antigen for use in immune targeting, leading to a major improvement in antigen affinity in comparison to an scFv. The often complex and highly structured nature of disease antigens renders the conjugation of a full IgG antibody impractical, making use of an scFab an attractive alternative. The poor binding of the anti-Clec9A scFv to plastic-bound or cell-surface Clec9A was potentially due to a lower intrinsic affinity and/or an unstable structure. In contrast, an scFab that includes the constant region of the light chain and first constant segment of the heavy chain, could be expressed and purified in isolation and was able to bind both plastic-bound Clec9A by ELISA and cell-surface Clec9A on transfected cells when fused to AD8 gp140. Despite this enhanced targeting ability in comparison to an scFv, the affinity of the scFab for Clec9A was lower than the full anti-Clec9A IgG. No binding was detected to Clec9A on a DC cell line, which displays a lower density of Clec9A than cells transiently transfected with a plasmid expressing Clec9A under the control of the strong CMV promoter. The scFab construct was also found to elicit high titers of anti-scFab antibodies, which may be immunodominant in comparison to the Env-specific antibody response. The absence of a consistent correlation between the anti-Env and anti-scFab antibody titres suggests the presence of the anti-scFab antibodies were not directly inhibiting the formation of anti-Env antibodies. The immunogenicity of the scFab moiety could be reduced by converting the constant regions of the scFab to equivalent murine sequences, which was not attempted in this proof of concept trial. However, the low binding of the scFab to Clec9A is likely insufficient to induce enhanced antibody titers following immunization, and effective targeting will likely require further improvement of this binding capacity.

The lower binding of the antibody fragments to Clec9A when compared to the native IgG may be a result of decreased stability, reduced affinity or reduced avidity. The former appeared to be the case for the scFv in particular, which was not able to be expressed on its own. Reduced scFv stability was not surprising, given this is known to be a potential disadvantage of scFvs, which are prone to misfolding, poor solubility or aggregation [80–83]. In contrast, Fabs and scFabs do not usually show the same tendencies [59, 84]. This was reflected in the results here, whereby the scFab was able to be expressed in isolation or fused to the C-terminus of Env and to bind both soluble Clec9A and membrane-bound Clec9A on transiently transfected cells. The presence of the entire light chain and C_H_1 region is thought to increase protein stability due to interactions between the variable and constant regions on each individual antibody chain, and between the constant regions, including the stabilizing disulfide bond [76]. The scFab is likely less stable–and therefore lower affinity–than the native IgG, which is able to bind Clec9A on the surface of DCs and displays significantly higher binding to the transiently transfected cells. Indeed, this phenomenon whereby IgGs display higher binding affinities compared to Fabs has been previously reported [85–87].

The bivalent nature of the IgG may also be contributing to a higher avidity interaction with Clec9A compared to the scFab, impacting its ability to target. While a single scFab binds antigen in a monovalent fashion, the trimeric nature of the Env should result in trivalent binding and improved avidity of the interaction with Clec9A. However, the relative positions of each scFab domain at the Env C-terminus is unknown, and may not permit multivalent Clec9A binding. While the linker between the Env and scFab is predicted to be 72Å in a fully extended state, it is only 36Å in a relaxed state [59], which may not allow for the scFabs to position apart by 117Å, the average distance between the Fab regions in a mouse IgG2a [88] (the isotype of the 10B4 antibody), suggesting the Env to scFab linker length may be too short to allow multivalent binding. Alternatively, the highly flexible nature of the linker may also result in the scFab moieties adopting non-optimal orientations for avid antigen binding. Improvements to the linking mechanism, such as a more rigid linker, achieved for example by a reduction in the proportion of glycine [89] or the use of a proline-rich sequence [90] may promote multivalent binding.

Despite the superior IgG affinity, the trimeric nature of Env renders conjugation of the full antibody difficult, thus a single chain targeting solution was sought. As the scFab demonstrates improved affinity compared to the scFv, further improvements to this targeting approach may be possible to enhance the affinity for Clec9A. One possible strategy is the stabilization of the scFab fragment by the addition of interdomain disulfide bonds, which have been shown to enhance the stability of scFv proteins [91, 92]. Alternatively, the use of targeting strategies not requiring the use of the scFab could be investigated. The targeting of Clec9A by a Clec9A-binding peptide, as has been previously demonstrated [93], may enable simpler conjugation to Env. Alternatively, the native IgG may be used if conjugated to Env via ‘Click Chemistry’ [94], or co-expressed with Env on a particulate immunogen such as a VLP or a nanoparticle, which have previously demonstrated Clec9A-targeting [95, 96]. These strategies would avoid the reduction in affinity arising from use of the scFab. An alternative anti-Clec9A antibody clone, such as those published previously [38], or targeting Env to an alternative surface molecule such as XCR1 [97, 98], CD11c [99] or CD180 [100] may form a more stable scFv or scFab, thus leading to higher affinity targeting. If an effective targeting mechanism is found, conjugation of an scFab to Env may have the additional advantage of reducing the immunodominance of the base-region of truncated Env that elicits mainly non-neutralizing antibodies during vaccination [101] through steric hindrance.

These data show that while scFab domains can be conjugated to Env without affecting the Env conformation, and can bind Clec9A, further improvements are required to improve the affinity and/or avidity of the Clec9A-binding interaction to enable efficient targeting *in vivo*. The elicitation of bNAbs by vaccination is elusive, and a strategy which enhances the formation of germinal centers and/or Tfh responses, which have been shown to enhance the elicitation of neutralizing antibodies [29, 30] may play a key role in overcoming this roadblock in HIV vaccine development. This, to our knowledge, is the first demonstration of an scFab conjugated to a pathogen-derived antigen for use in immune targeting, with implications beyond HIV vaccine design. The improved affinity of the scFab in comparison to an scFv suggests that the use of an scFab for immune-targeting during immunization or for cancer immunotherapy warrants further investigation and presents an attractive alternative where scFv targeting has been unsuccessful.

## Supporting information

S1 Fig Binding of Clec9A-targeting constructs to Clec9A on a Dendritic Cell line. The DC cell line Mutu DC 1940 was stained with either the AD8 constructs or the 10B4 anti-Clec9A mAb. (A) Forward and side scatter gates were used to select dendritic cells, and single cells, followed by selection of propidium iodide negative cells to exclude dead cells. (B) Histogram showing PE fluorescence, used to detect binding of the 10B4 mAb or an isotype control, or CF647 fluorescence, detecting binding of Env constructs. Cells were gated as described in A and curves were normalized to mode.
(TIF)

S2 Fig Reactivity of sera to Envs of diverse Clades. Env (either AD8 SOSIP v4.1 (SOSIP version of vaccine antigen, clade B), BG505 SOSIP v2 (clade A), SC45 SOSIP v4.1 (clade B) and MW uncleaved gp140 (clade C)) were used to coat ELISA plates, then mouse sera were diluted in a half-log series from an initial 1:100 dilution. Titers were defined as the dilution at which the ELISA curve was equal to 5x the average of the adjuvant only group. The coating antigen is given in the title for each graph, while the x-axis shows the different vaccination groups. Graphs show the mean ± SEM of two independent experiments.
(TIF)

S1 Data Raw data values for the presented figures. Raw numerical data used to generate Figs 1B, 1D, 2C, 3, 5B and 5C.
(XLSX)
