# Supplementary figures and images for: Conjugation of an scFab domain to the oligomeric HIV envelope protein for use in immune targeting
Source: PLoS One. 2019 Aug 20;14(8):e0220986. doi: 10.1371/journal.pone.0220986 (PMC6701830; doi:10.1371/journal.pone.0220986)

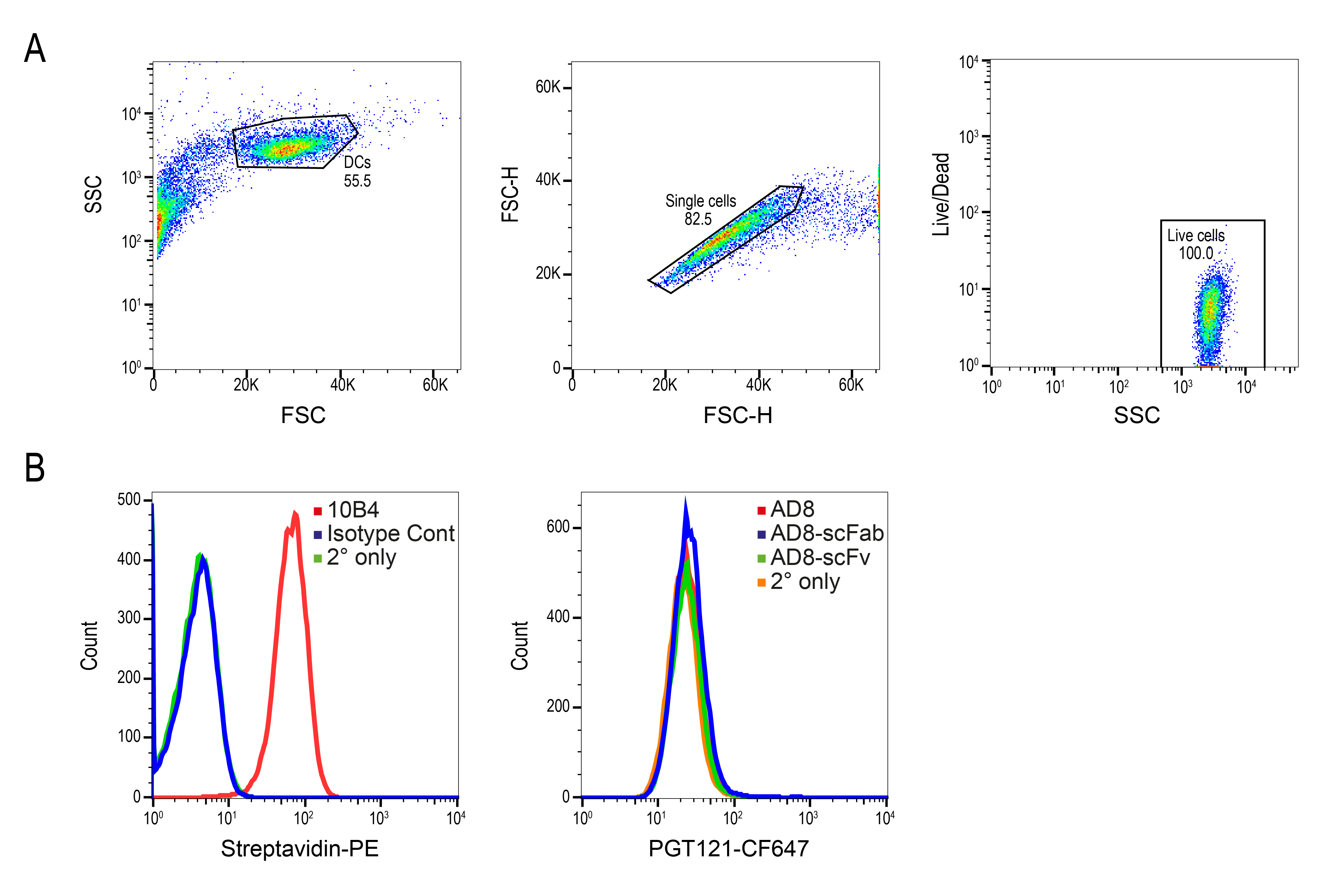

Supplement: S1 Fig — The DC cell line Mutu DC 1940 was stained with either the AD8 constructs or the 10B4 anti-Clec9A mAb. (A) Forward and side scatter gates were used to select dendritic cells, and single cells, followed by selection of propidium iodide negative cells to exclude dead cells. (B) Histogram showing PE fluorescence, used to detect binding of the 10B4 mAb or an isotype control, or CF647 fluorescence, detecting binding of Env constructs. Cells were gated as described in A and curves were normalized to mode. (TIF) [file pone.0220986.s001.tif]

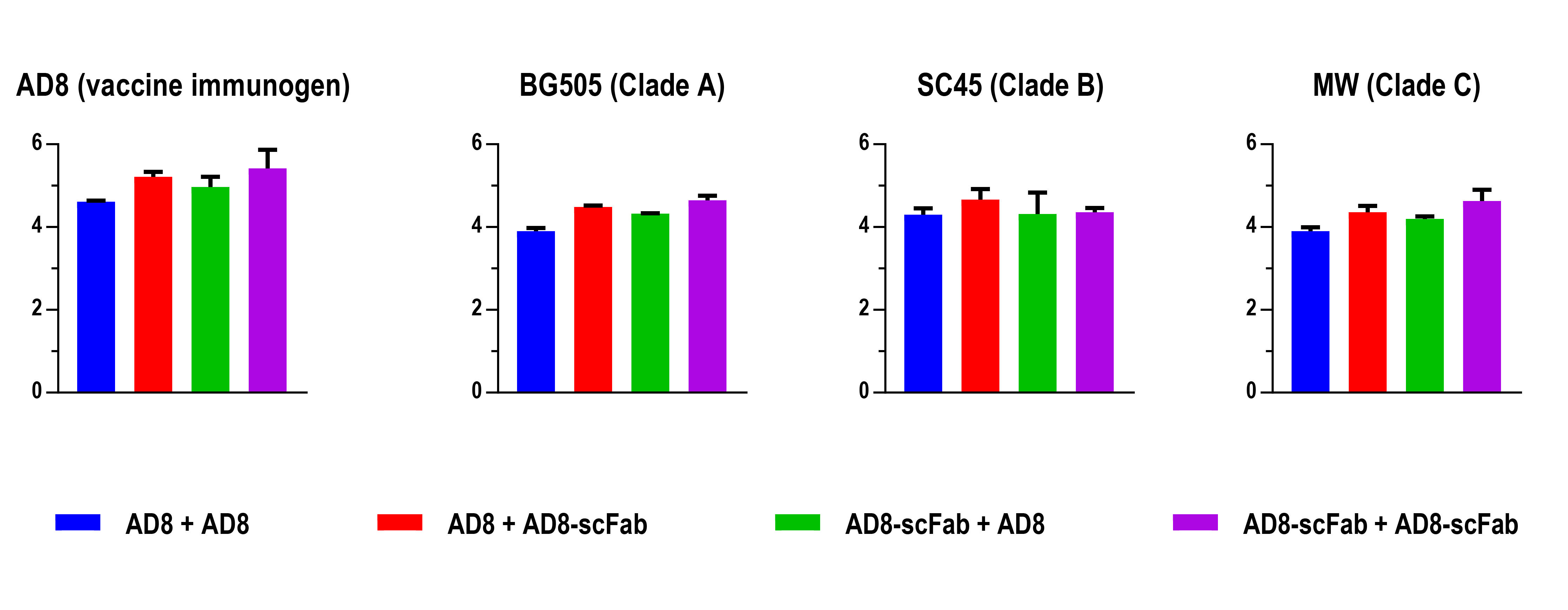

Supplement: S2 Fig — Env (either AD8 SOSIP v4.1 (SOSIP version of vaccine antigen, clade B), BG505 SOSIP v2 (clade A), SC45 SOSIP v4.1 (clade B) and MW uncleaved gp140 (clade C)) were used to coat ELISA plates, then mouse sera were diluted in a half-log series from an initial 1:100 dilution. Titers were defined as the dilution at which the ELISA curve was equal to 5x the average of the adjuvant only group. The coating antigen is given in the title for each graph, while the x-axis shows the different vaccination groups. Graphs show the mean ± SEM of two independent experiments. (TIF) [file pone.0220986.s002.tif]
